# Increased Occurrence of Valproic Acid-Induced Hyperammonemia in Carriers of T1405N Polymorphism in Carbamoyl Phosphate Synthetase 1 Gene

**DOI:** 10.1155/2013/261497

**Published:** 2013-08-07

**Authors:** Piotr K. Janicki, Dmitri Bezinover, Marek Postula, Robert S. Thompson, Jayant Acharya, Vinita Acharya, Cathy McNew, J. Daniel Bowman, Iwona Kurkowska-Jastrzebska, Dagmara Mirowska-Guzel

**Affiliations:** ^1^Laboratory of Perioperative Genomics and Anesthetic Preoperative Evaluation Clinic, Department of Anesthesiology, Pennsylvania State University College of Medicine, MS Hershey Medical Center, H187, 500 University Drive, Hershey, PA 17033, USA; ^2^Department of Experimental and Clinical Pharmacology, Warsaw Medical University, Krakowskie Przedmiescie 26/28, 00-927 Warsaw, Poland; ^3^Department of Neurology, Pennsylvania State University MS Hershey Medical Center, H187, 500 University Drive, Hershey, PA 17033, USA; ^4^2nd Department Neurology, National Institute of Psychiatry and Neurology, Ulica Sobieskiego 9, 02-957 Warsaw, Poland

## Abstract

Numerous cases of severe and life-threatening hyperammonemia (HA) related to the treatment of epileptic seizures with valproic acid (VPA) have been previously reported in the medical literature. The aim of this prospective, multicenter study was to verify the putative association between T1405 polymorphism and occurrence of VPA-induced HA in the cohort of 142 adult Caucasian patients with epilepsy treated with VPA for at least 1 year and with normal liver functions. The nonsynonymous T1405N polymorphism genotyping was performed by real-time TaqMan PCR genotyping. In addition to plasma ammonia level, concentrations of liver enzymes and total VPA were measured in plasma with standard laboratory methods. HA (defined as ammonia plasma level >65 **μ**mol/L) was observed in total of 11 (7.7%) of patients treated with VPA, and the carrier status for the investigated polymorphism was significantly (*P* = 0.009, odds ratio 5.4 with 95% confidence interval of 1.58–18.43) associated with the occurrence of HA. The results of this study support a notion that in the Caucasian patients with epilepsy undergoing VPA therapy, a T1405N (4217C > A, rs1047891) nonsynonymous variant was a significant risk factor for the occurrence of HA, even in patients with normal plasma levels of VPA.

## 1. Introduction

Valproic acid (VPA) has a clinical use as an anticonvulsant and mood-stabilizing drug, primarily in the treatment of epilepsy, bipolar disorder, and, less commonly, major depression. Hyperammonemia (HA) has been generally associated with liver failure or rare genetic mutations in enzymes participating in the urea cycle. However, in some patients treated with VPA, severe HA has been observed in the absence of liver failure and resulting in vomiting, ataxia, behavioral changes, lethargy, somnolence, or, in extreme cases, coma. Numerous cases of severe and life-threatening HA related to the VPA treatment mostly in children, adolescents, and also adults [1] have been previously reported in the medical literature (*>220 published reports in Medline since 1980 by search using VPA and HA as keywords in August 2012*). The exact mechanism of VPA-induced encephalopathy is unclear but relates to the accumulation of toxic VPA metabolites and elevated ammonia levels [2, 3].

The prior limited data from exclusively Japanese cohort suggested that patients treated with VPA and who carry the T1405N (4217C > A, rs1047891) missense single nucleotide polymorphism (SNP) in the carbamoyl phosphate synthetase 1 gene might be more likely to experience HA [4]. The main goal of this project was to investigate, for the first time in Caucasian cohort, if carbamoyl phosphate synthetase 1 (CPS1) gene T1405N missense variant can be associated with increased occurrence of HA in subjects treated with VP.

## 2. Materials and Methods

### 2.1. Patients and Sample Collection

This prospective multicenter cohort study was performed between April 2011 and September 2012 using the study protocol which was approved by IRB at PSU Hershey Medical Center, Hershey, PA, USA, and Institute of Psychiatry and Neurology in Warsaw, Poland. Written informed consent was obtained from all patients. All subsequent Caucasian adult patients treated with VPA (irrespective of age and gender) and satisfying the inclusion and exclusion criteria were enrolled into the study. Inclusion criteria included adults (>18 years of age) and treatment with VPA for the period of >1 year. Exclusion criteria included pregnancy, severe hepatic disease (as documented by both clinical diagnosis and grossly abnormal liver function tests), end-stage kidney disease, that is, requiring dialysis, and advance mental retardation that prevents signing of the informed consent. HA was defined as the plasma ammonia level >65 *μ*mol/L (representing values larger than 95 percentile of the normal range by standard laboratory testing) at the time of enrollment.

### 2.2. Genetic Analysis

Whole blood samples were collected into EDTA-tubes and frozen at −80°C until analysis. Genomic DNA (gDNA) was extracted from blood sample employing membrane ultrafiltration method using Fuji MiniGene 80 semiautomatic extractor (Fujifilm Life Sciences distributed by Autogen, Holliston, MA, USA), according to the manufacturer's recommendations. The saliva samples were collected into Oragene containers (DNA Genotek, Canada) and extracted according to the manufacturer's recommendations. gDNA analysis of clinical samples was performed in triplicates using custom SNP real-time PCR TaqMan genotyping assay (Applied Biosystems, Foster City, CA, USA) and according to the manufacturer's protocol. Briefly, gDNA was incubated with two custom designed flanking primers for amplifying the sequence of interest and two TaqMan MGB probes for detecting specific alleles containing a reporter dye (VIC and FAM) at the 5′ end of each allele-specific probe and nonfluorescent quencher at the 3′ end of the probe. The real-time PCR analysis was performed using TaqMan Universal Master Mix, No AmpErase UNG in 384-well optical plate using an Applied Biosystems Prism 7900HT Sequence Detection System at PSHMC Genomic Core Facilities, Hershey, PA, USA. Following PCR amplification, endpoint fluorescence measurements from each well will be obtained, and the alleles present in each sample were identified. 

### 2.3. Statistical Analysis

Hardy-Weinberg equilibrium for analyzed genotypes was evaluated using Fisher's Exact test. The primary analysis for the first specific aim utilized univariate logistic regression with a carrier status for minor allele as independent variable and a presence of HA (yes/no) phenotype as dependent variable. The logistic regression procedure enabled us to estimate the log of the odds ratio (OR), a measure of the increase in odds of experiencing HA for subjects carrying the variant compared to the wild-type subjects. We obtained 95% confidence interval around this estimate and the *P* value for the OR. The *P* value was compared with the predefined cutoff for statistical significance (alpha = 0.05). A significant association between carrying T1405N variant and increased risk of VPA-induced HA has been expected, therefore, the association was adjusted for the predefined covariates to determine whether the association between CPS1 4217C > A and risk of VPA-induced HA remains significant after inclusion of factors. Given the expected population incidence of VPA-induced HA of approximately 10%, expected frequency for minor allele of 0.35, alpha level = 0.05, and the power of 0.8, at least 140 patients were needed to detect OR >4 for experiencing HA in carriers of minor allele.

## 3. Results

In total, 142 subjects were enrolled in the study, from which 97 were enrolled in Hershey, PA, USA, and 45 in Warsaw, Poland. In 95% of the enrolled patients, VPA was used as a primary agent to treat epilepsy. The clinical characteristics of the patients are summarized in Table 1. The observed plasma level of ammonia ranged between 9 and 181 *μ*mol/L (mean ± standard deviation 31.6 ± 16.4 *μ*mol/L) in the investigated cohort of patients, and HA (defined as ammonia plasma level >65 *μ*mol/L) was observed in total of 11 (7.7%) of patients treated with VPA. None of the patients presented with HA was symptomatic during enrollment period.

The distribution of CPS1 genotypes for the polymorphism within overall study population fulfilled Hardy-Weinberg criteria (Fisher's Exact test, *P* > 0.05). The observed genotype distribution (CC denotes homozygosity for the C-encoded Thr1405 variant, AA homozygosity for the A-encoded Asn1405 variant, and AC heterozygosity for polymorphism at position 1405) in investigated patients was CC : CA : AA = 69 : 61 : 13, *N* = 142). The minor allele (A) frequency was 0.307 (Table 2). No significant differences among the two centers were observed in genotype distribution or clinical characteristics in patients enrolled in the study with exception of statistically significant more patients on multiple antiepileptic medications in Pennsylvania cohort and higher level of ammonia in the Warsaw cohort (Table 1).

**Figure 1:**
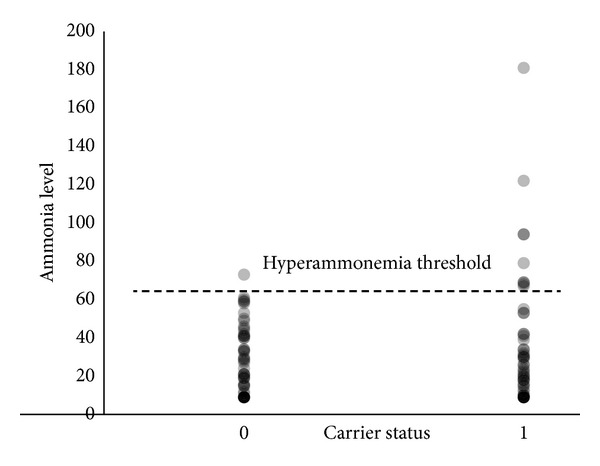
Distribution of individual blood ammonia concentrations at the time of enrollment according to patients carrier status for minor allele in T1405N polymorphism (noncarriers = 0, carriers = 1). Vertical axis—blood ammonia concentrations in *μ*mol/L. The horizontal line denotes the threshold level of diagnosis of hyperammonemia.

The univariate analysis of the carriers and noncarriers of the Thr1405 variant revealed a significant association with HA phenotype in the overall study group (*P* = 0.009, odds ratio 5.4 with 95% confidence interval of 1.58–18.43). Multifactorial logistic regression indicated that Thr1405 carrier status is independently associated with HA after adjusting for study center, age, gender, total 24 hr VPA dose, VPA plasma concentrations, and the number of concomitant antiepileptic medications administered with VPA (not shown). No significant differences between medians of absolute blood concentrations of ammonia were observed in the three genotypes for the investigated variant (Figure 1) or carrier versus noncarriers of the investigated minor allele. 

## 4. Discussion

Identifying early biomarkers for patients at risk for VPA-induced HA remains an elusive research goal. Both heterozygous and homozygous carrier states of the T1405N polymorphism and concomitant administration of two or more anticonvulsants with VPA were shown previously to be independent risk factors for developing HA in the relatively small cohort of patients (*N* = 79) from Japan and single case report from our laboratory [4, 5]. In the recent larger study, Yamamoto et al. identified several other risk factors for HA in PWE, including VPA dose and use of hepatic enzyme inducers, although genetic polymorphism was not analyzed in this study [6]. Built on the previous reports, we hypothesized that in a Caucasian population of patients without liver failure and treated with VPA, genetically determined variations in CPS1 function would be associated with increased incidence of HA. Our data confirms this hypothesis, because we did observe that the percentage of patients with HA was significantly higher in the carriers of the minor allele when compared with the carriers of two wild-type alleles for the investigated polymorphism (i.e., dominant model of inheritance). Because the current study was not powered to investigate other factors involved in HA, we cannot conclude with absolute certainty that other factors (e.g., VPA dose, concomitant use of other anticonvulsants) could be associated with increased occurrence of HA in the investigated cohort of patients during prolonged VPA treatment. 

The urea cycle is an essential process for the removal of ammonia from the body, and its initial step is the conversion of ammonia and bicarbonate into carbamoyl phosphate via hepatic mitochondrial CPS1. The effects of VPA on urea metabolism are complex and occur in the liver mitochondria. Inherited deficiency of CPS1 with severe HA is a rare autosomal recessive disorder with a prevalence of 1 : 200,000–1 : 800,000 (http://omim.org/entry/237300). At present, more than 60 severe deficiency-causing mutations in the CPS1 gene have been identified (2012 Human Genome Mutation Database, http://www.hgmd.org/). Less severe deficiency or reduced activity of CPS1 is thought to be associated with VPA-induced HA. VPA preferentially inhibits CPS1, which in turn may lead to a dose independent increase in the concentration of its substrate ammonia in the blood [7, 8]. VPA may also inhibit carnitine transportation into the mitochondria which is responsible for the shift in the balance of metabolism towards protein degradation with subsequent HA. T1405 polymorphism in the CPS1 gene results in a threonine to asparagine amino acid substitution at an important cofactor (i.e., N-acetylglutamate) binding site in exon 36 of CPS1 [9]. Previous studies demonstrated the association of the CPS1 T1405 polymorphism with neonatal pulmonary hypertension and necrotizing enterocolitis [10–12].

Several studies reported that asymptomatic HA patients had problems related to seizure control, developmental delay, and intellectual disability [1–3]. In addition, it was reported recently that the preexisting HA may be further exacerbated in stress-related situations (perioperative period for surgical procedures) which may increase the postoperative risk for these patients [13, 14]. It is important therefore to establish risk factors for an elevated ammonia level and to perform the measurement of ammonia irrespective of whether patients are asymptomatic or not, in particular in situations which may lead to further exacerbation of HA. 

In conclusion, the T1405N (4217C > A, rs1047891) missense was found to be a significant risk factor for the occurrence of HA in Caucasian patients undergoing VPA therapy, even in subjects with normal plasma levels of VPA.

## Figures and Tables

**Table 1:** Demographic and clinical characteristics of Caucasian patients treated with valproic acid (VPA) enrolled into the study protocol.

	Pennsylvania cohort (*N* = 97)	Warsaw cohort (*N* = 45)	Combined (*N* = 142)
Minor allele (A) frequency in CPS1 4217C > A	0.302	0.318	0.307
Number of carriers of the minor allele (%)	50 (52%)	23 (52%)	73 (52%)
Gender (F/M)	49/48	22/23	71/71
Age (years)	44.3 ± 15.1	51.3 ± 19.4	48 ± 17
Weight (kg)	87.5 ± 19.9	76.7 ± 17.7	82 ± 18
Number of patients administered multiple antiepileptic drugs (%)	56 (57.7%)*	11 (24.4%)	67 (47.2%)
Number of subjects with hyperammonemia (>65 *μ*mol/L) (%)	5 (5.2%)	6 (13.3%)	11 (7.7%)
VPA dosage (mg/24 hrs)	1109 ± 402	950 ± 378	1045 ± 398
Plasma VPA level	78 ± 32	65 ± 21	72 ± 27
Aminotransferases (U/L):			
AST	33 ± 16	22 ± 12	28 ± 14
ALT	24 ± 15	21 ± 17	23 ± 16
Blood ammonia level	24 ± 21*	39 ± 28	32 ± 16
(*μ*mol/L): average ± SD, median (interquartile range) and data range	22 (19–29)* 113	37 ( 35–55) 162	24 (11–76) 175

*P < 0.05 by *T* test or *P* < 0.05 by Mann-Whitney test for differences between investigated cohorts.

**Table 2:** Distribution of CPS1 genotypes for the T1405N polymorphism in patient with normal ammonia level and patients with hyperammonemia (HA, defined as ammonia plasma level >65 *µ*mol/L).

	CC genotype	CA genotype	AA genotype	Numbers of carriers of minor A allele (percentage)	Minor allele frequency
Patient with normal ammonia level (*N* = 131)	68 (52%)	54 (41%)	10 (7%)	64 (45%)	0.28
Patients with HA (*N* = 11)	1 (9%)	7 (64%)	3 (27%)	10 (91%)*	0.6

Results are expressed as absolute numbers of patients (percentage). CC denotes homozygosity for the C-encoded Thr1405 variant, AA homozygosity for the A-encoded Asgn1405 variant, and CA heterozygosity for this polymorphism. *P = 0.009 by logistic regression and Fisher's exact test.
